# Feasibility and image quality of bright-blood and black-blood phase-sensitive inversion recovery (BOOST) sequence in clinical practice using for left atrial visualization in patients with atrial fibrillation

**DOI:** 10.1007/s00330-023-10257-3

**Published:** 2023-10-07

**Authors:** Zsófia Dohy, Máté Kiss, Ferenc Imre Suhai, Karl Kunze, Radhouene Neji, Gábor Orbán, Zsófia Drobni, Csilla Czimbalmos, Vencel Juhász, Liliána Szabó, Rene Botnar, Claudia Prieto, Béla Merkely, Nándor Szegedi, Hajnalka Vágó

**Affiliations:** 1 Heart and Vascular Centre, Semmelweis University, 68 Varosmajor St, Budapest, H-1122 Hungary; 2 Siemens Healthcare Hungary, Budapest, Hungary; 3 Siemens Healthcare Limited, Frimley, UK; 4 School of Biomedical Engineering and Imaging Sciences, King’s College London, London, UK

**Keywords:** Magnetic resonance angiography, Atrial fibrillation, Pulmonary veins, Image quality, Three-dimensional imaging

## Abstract

**Objectives:**

Visualizing left atrial anatomy including the pulmonary veins (PVs) is important for planning the procedure of pulmonary vein isolation with ablation in patients with atrial fibrillation (AF). The aims of our study are to investigate the feasibility of the 3D whole-heart bright-blood and black-blood phase-sensitive (BOOST) inversion recovery sequence in patients with AF scheduled for ablation or electro-cardioversion, and to analyze the correlation between image quality and heart rate and rhythm of patients.

**Methods:**

BOOST was performed for assessing PVs both with T2 preparation pre-pulse (T2prep) and magnetization transfer preparation (MTC) in 45 patients with paroxysmal or permanent AF scheduled for ablation or electro-cardioversion. Image quality analyses were performed by two independent observers. Qualitative assessment was made using the Likert scale; for quantitative analysis, signal to noise ratios (SNR) and contrast to noise ratios (CNR) were calculated for each PV. Heart rate and rhythm were analyzed based on standard 12-lead ECGs.

**Results:**

All MTC-BOOST acquisitions achieved diagnostic quality in the PVs, while a significant proportion of T2prep-BOOST images were not suitable for assessing PVs. SNR and CNR values of the MTC-BOOST bright-blood images were higher if patients had sinus rhythm. We found a significant or nearly significant negative correlation between heart rate and the SNR and CNR values of MTC-BOOST bright-blood images.

**Conclusion:**

3D whole-heart MTC-BOOST bright-blood imaging is suitable for visualizing the PVs in patients with AF, producing diagnostic image quality in 100% of cases. However, image quality was influenced by heart rate and rhythm.

**Clinical relevance statement:**

The novel 3D whole-heart BOOST CMR sequence needs no contrast administration and is performed during free-breathing; therefore, it is easy to use for a wide range of patients and is suitable for visualizing the PVs in patients with AF.

**Key Points:**

*• The applicability of the novel 3D whole-heart bright-blood and black-blood phase-sensitive sequence to pulmonary vein imaging in clinical practice is unknown.*

*• Magnetization transfer-bright-blood and black-blood phase-sensitive imaging is suitable for visualizing the pulmonary veins in patients with atrial fibrillation with excellent or good image quality.*

*• Bright-blood and black-blood phase-sensitive cardiac magnetic resonance sequence is easy to use for a wide range of patients as it needs no contrast administration and is performed during free-breathing.*

**Supplementary Information:**

The online version contains supplementary material available at 10.1007/s00330-023-10257-3.

## Introduction

Imaging of the left atrium (LA) in atrial fibrillation (AF) can provide insights into the etiology and risk stratification and influences the therapeutic management of patients. Assessment of LA function and structure has a predictive value in the occurrence of AF and the risk of stroke [1]. Thromboembolism is the most severe complication of AF. To exclude LA appendage thrombus before an electro-cardioversion or ablation therapy of AF is particularly important [2, 3]. Visualizing LA anatomy including the pulmonary veins (PVs) is important for planning the procedure of pulmonary vein isolation with ablation [4]. The PV anatomy is conventionally assessed before ablation using computer tomography or 3 dimensional (3D) contrast-enhanced cardiac magnetic resonance (CMR) imaging [5, 6].

CMR imaging is an excellent method for evaluating cardiac anatomy and function. 3D contrast-enhanced CMR angiography (CMRA) is applicable for the assessment of LA dimensions and PV anatomy, whereas 2D cardiac cine imaging is useful for evaluating LA function. Contrast-enhanced CMRA acquisitions are performed during the patient’s breath-holding and are taken in time with the first-pass of a contrast agent. Inadequate timing of image acquisition or problems with the patient’s breath-holding may result in poor image quality. Another option is a contrast-enhanced electrocardiographic (ECG) and respiratory-gated 3D (balanced steady-state free precession (bSSFP) or gradient echo (GRE)) sequence, which helps to depict the pulmonary veins even if the patient is unable to hold their breath [7]. The biggest downside of this technique is the quite long and unpredictable acquisition time which highly depends on the patient’s breathing pattern. Due to the long scan time, this acquisition cannot be repeated when the image quality is poor.

A novel MRI sequence, a 3D whole-heart bright-blood and black-blood phase-sensitive (BOOST) inversion recovery sequence that needs no contrast administration and is performed during free-breathing using image-based navigation, has been introduced recently [8, 9]. One of the biggest advantages of BOOST is that it acquires 2 differently weighted bright-blood volumes in an interleaved fashion. The two bright-blood images can be combined using a subtraction-based approach (iT2prep) [10] or a phase-sensitive inversion recovery (PSIR)–like reconstruction in order to provide both bright- and black-blood volumes. Initial validation has proven the clinical applicability of the technique, showing that simultaneous bright- and black-blood whole-heart MRI with T2 preparation (T2prep) can help to identify the coronary lumens and thrombi [9]. The same study tested the feasibility of BOOST for simultaneous black-blood late gadolinium enhancement (LGE) assessment and bright-blood coronary angiography [11]. Additionally, magnetization transfer (MTC)–prepared BOOST (MTC-BOOST) is possible for the imaging of PVs, which provides excellent image quality and offers good depiction of the intracardiac and vascular anatomy at congenital heart disease patients which is comparable to the standard T2prep bSSFP sequence, as shown by earlier studies [12]. MTC-BOOST was compared with the standard T2prep bSSFP technique in pulmonary vein visualization, where MTC-BOOST overperformed the standard technique. This study was based on the examination of 11 healthy subjects and only 4 patients with AF [8] and they concentrated to present the advantages of non-rigid motion-corrected algorithms. Nevertheless, there is limited data available on the applicability of the BOOST sequence in clinical practice based on studies of larger numbers of patients.

The aims of our study are to investigate the feasibility of the BOOST sequence in patients with AF scheduled for ablation or electro-cardioversion, analyze the image quality with both quantitative and qualitative methods, and compare the results with those patients who had sinus rhythm. Finally, we want to investigate the correlation between image quality and heart rate and rhythm of patients.

## Methods

### Patients

Informed consent was obtained from each patient. Ethical approval was obtained from the Medical Research Council (IV/4962-3/2021/EKU), and this study was performed in accordance with the ethical standards in the 1964 Declaration of Helsinki and its later amendments.

Fifty-four patients with atrial tachyarrhythmias (paroxysmal or persistent AF, atrial flutter, atrial tachycardia) scheduled for ablation or electro-cardioversion in the Heart and Vascular Center of Semmelweis University were prospectively involved in the study between May 2021 and December 2021. Patients with pacemakers or implanted cardioverter defibrillators or with any contraindications of CMR examination were not involved in the study. Nine patients were excluded because CMR study was incomplete (MTC or T2prep images were missing). All together 45 patients were involved the study. Patients’ clinical demographic and clinical characteristics are summarized in Table 1.

**Table 1:** Summary table of the demographic and clinical characteristics of the study population

Total number of patients	45
Male sex, *N* (%)	25 (56%)
Age mean ± standard deviation	64 ± 10 years
**Clinical diagnosis**	*N* (%)
Paroxysmal atrial fibrillation	32 (71%)
Persistent atrial fibrillation	6 (13%)
Atrial tachycardia	3 (7%)
Atrial flutter	4 (9%)
**Therapeutic intervention**	*N* (%)
Ablation	39 (87%)
Electro-cardioversion	6 (13%)
**Rhythm at the time of CMR**	*N* (%)
Sinus rhythm	30 (67%)
Atrial fibrillation	12 (27%)
Atrial tachycardia	1 (2%)
Atrial flutter	2 (4%)
**Patients history**	*N* (%)
Previous ablation	4 (9%)
Hypertension	33 (73%)
Diabetes mellitus	8 (18%)
Dilated cardiomyopathy	1 (2%)
Ischemic heart disease	6 (13%)
Thyroid disorder	7 (16%)

A standard 12-lead ECG (25 mm/s and 10 mm/mV) was obtained within 1 h before or after the CMR examination while all patients were in a supine position during quiet respiration. Thirty patients had sinus rhythm, 12 patients had AF, two patients had atrial flutter, and one patient had atrial tachycardia at the time of the CMR examination. Patients with AF, atrial flutter, and atrial tachycardia were analyzed together as a group of patients with atrial tachyarrhythmias.

### CMR protocol

**Fig. 1:**
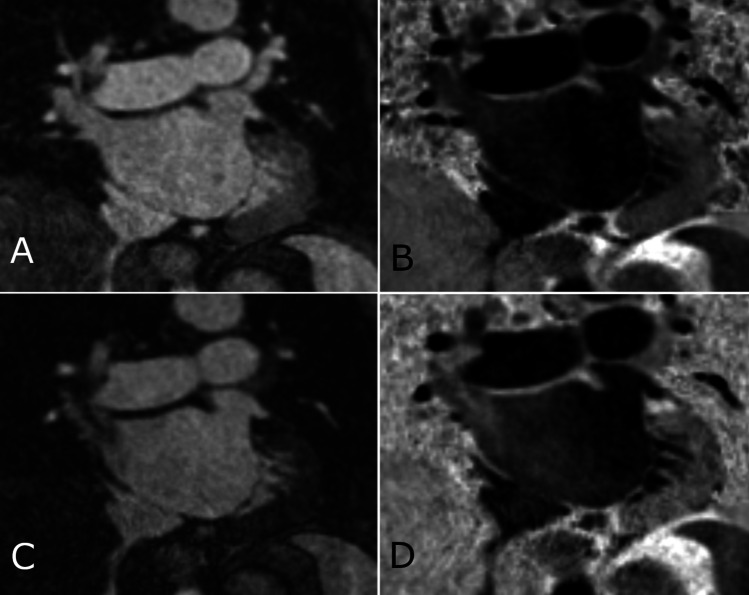
BOOST imaging of the left atrium. **A** Bright-blood magnitude image of MTC-BOOST. **B** Black-blood PSIR image of MTC-BOOST. **C** Bright-blood magnitude image of T2prep BOOST. **D** Black-blood PSIR image of T2prep BOOST. Excellent image quality of MTC-BOOST (**A**, **B**) can be observed. T2prep images (**C**, **D**) show the often observed inflow artifact in the right superior pulmonary vein

CMR examinations were performed on a 1.5-T MR scanner (MAGNETOM Aera, Siemens Healthcare). Two versions of an ECG-triggered 3D whole-heart bright-blood and black-blood bSSFP (BOOST) prototype sequence were performed independently from each other in direct succession: the first with a T2 preparation module (T2prep) and the second with an MT preparation (MTC) in combination with an adiabatic inversion pulse as previously described [8, 9]. Image navigation (iNAV) was used for respiratory motion estimation and compensation which allows measurements to be taken under free-breathing conditions and ensures 100% scan efficiency and predictable scan time [13]. In order to define an acquisition window during the cardiac cycle with the least motion, we applied an automated cardiac resting-phase detection algorithm [14], which was based on a free-breathing 4-chamber view acquired previously. The image acquisition window duration was set to 100 ms for each patient and placed according to the position indicated by the automatic resting-phase detection after being checked by the examining physician. All BOOST measurements were performed with the coverage of LA including the ostia of PVs and the appendage. The non-rigid motion-corrected magnitude T2Prep inversion recovery (IR) BOOST and reference T2Prep BOOST volumes were combined in a PSIR reconstruction [15] to obtain a PSIR BOOST black-blood volume [11]. Similarly, the magnitude MTC-IR BOOST and reference MTC-BOOST volumes were combined in a PSIR-like reconstruction to obtain a black-blood PSIR BOOST (Fig. 1) [8]. Imaging parameters are summarized in Supplementary Table S1.

### Image analysis

**Fig. 2:**
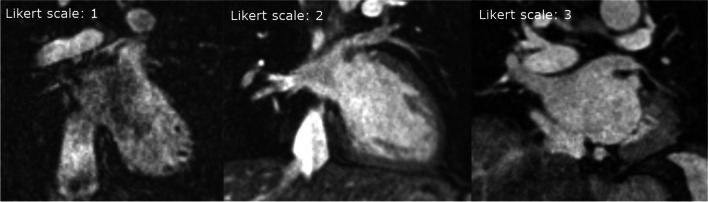
Qualitative image quality analysis of BOOST images with the Likert scale. 1 point: poor image quality, PVs are not detected (a representative case of T2prep magnitude bright-blood image with poor quality because of significant inflow artifacts in the pulmonary veins and in the atrium), 2 points: good image quality, artifacts in the PV(s) (a representative case of MTC magnitude bright-blood image with good quality, mild motion artifacts in the PVs), and 3 points: excellent image quality, no artifacts (a representative case of MTC magnitude bright-blood image with excellent quality)

Image quality analyses were performed by two independent observers (Obs1: F. I. Suhai and Obs2: M. Kiss) who were blinded to the heart rate and rhythm of patients. Qualitative assessment was made using the Likert scale as follows: 1 point indicated poor image quality, PVs are not detected; 2 points indicated good image quality, artifacts in the PV(s); and 3 points indicated excellent image quality, no artifacts (Fig. 2).

For quantitative analysis, regions of interest (ROI) were signed in all four PVs to measure signal intensities (SI). The mean background SI and the standard deviation (SD) of background SI were measured in the lung tissue. Signal to noise ratios (SNR) were calculated in each PV: mean SI of a PV / SD of background SI. Contrast to noise ratios (CNR) were also calculated in each PV: absolute value of (mean SI of a PV − mean background SI) / SD of background SI.

The image quality analyses were performed both on the bright-blood magnitude and black-blood PSIR images.

### Statistical analysis

Continuous data are expressed as the mean ± standard deviation (SD). The normality of the distribution of the data was investigated with the Shapiro-Wilk test. Group characteristics were compared with an independent *t*-test or the Mann-Whitney test, as appropriate. The correlation between heart rate and the SNR and CNR values was calculated with Spearman’s correlation analysis. The interobserver agreement was examined with the intraclass correlation coefficient (ICC score). Differences were considered statistically significant when *p* < 0.05. All analyses were performed by using MedCalc software (version 17.9.5).

## Results

### Image quality of T2prep BOOST

**Fig. 3:**
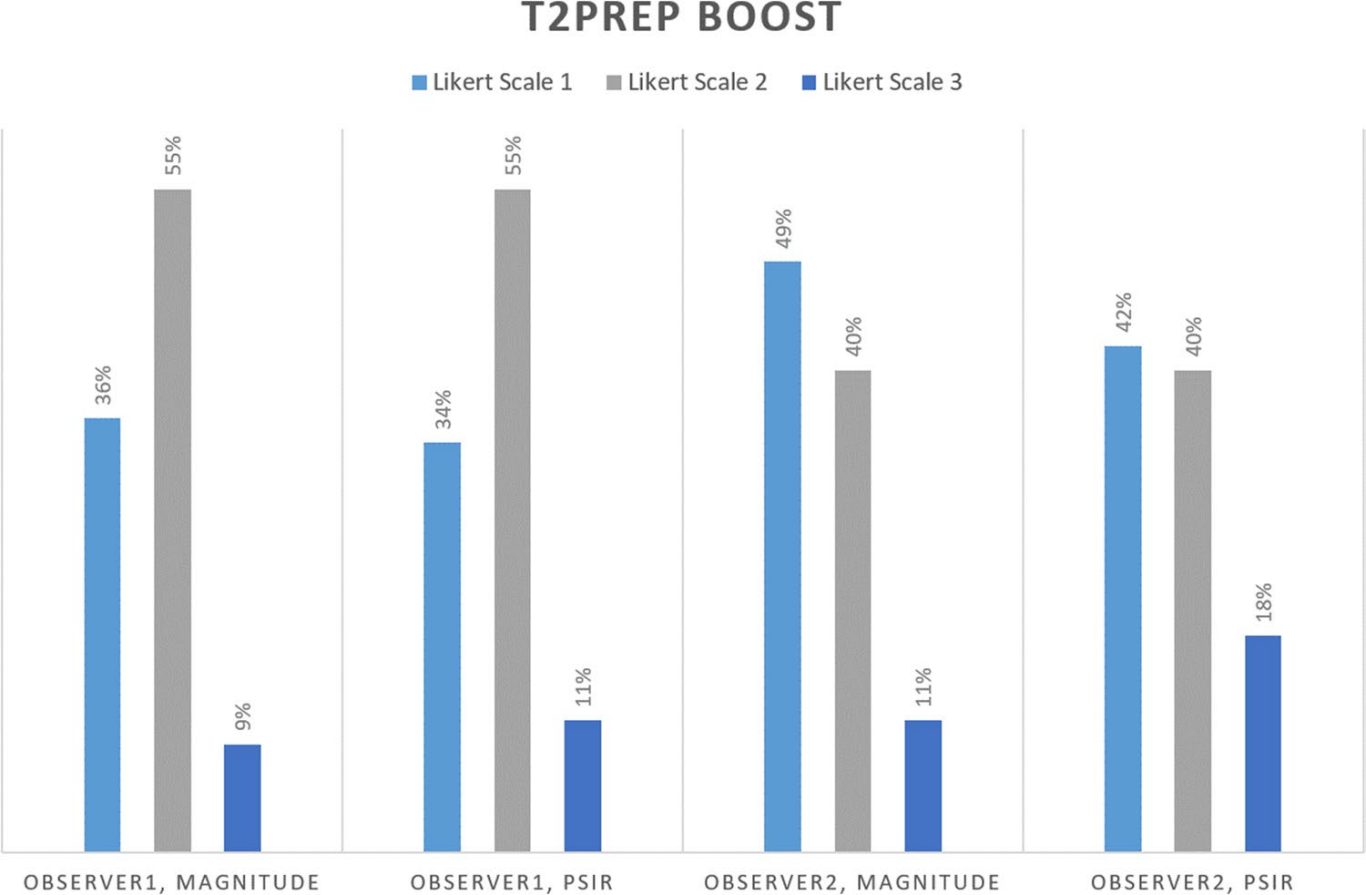
Qualitative assessment of image quality of T2Prep BOOST. Proportions of image quality ratings of magnitude and PSIR images by the two observers

A significant proportion of T2prep BOOST images were not suitable for assessing PVs, or artifacts were in the PVs. Only a small proportion of the images had excellent image quality without artifacts in the PVs (Fig. 3). Because of this result, further quantitative image quality analysis was not performed on the T2prep BOOST images.

### Qualitative assessment of image quality of MTC-BOOST

**Fig. 4:**
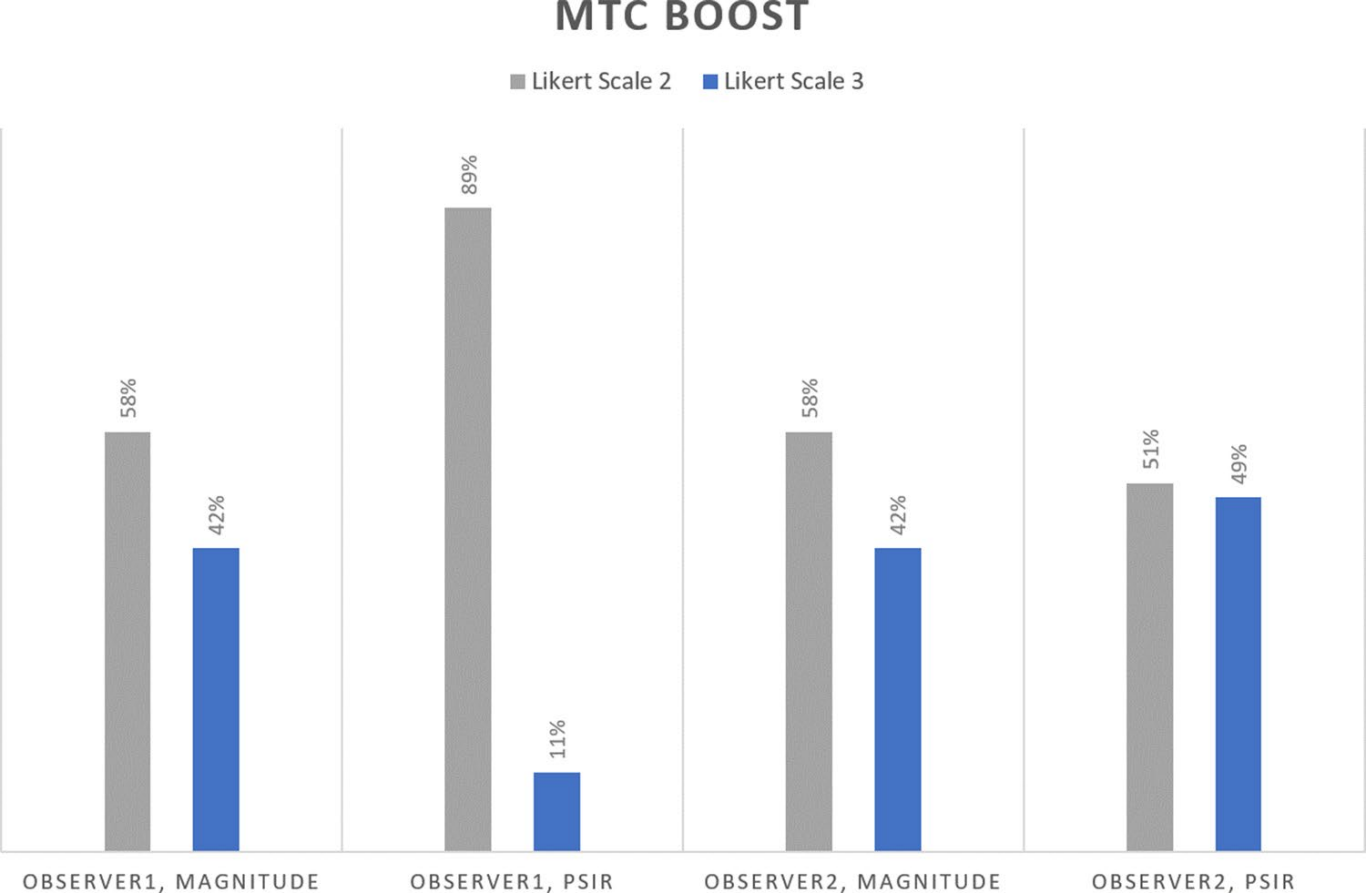
Qualitative assessment of image quality of MTC-BOOST. Proportions of image quality ratings of magnitude and PSIR images by the two observers

Among the MTC-BOOST images examined, there were no images of poor quality in which the PVs could not be adequately assessed. Both observers rated 42% of the bright-blood magnitude images as excellent image quality without artifacts (Likert scale 3) and 58% as good image quality with artifacts (Likert scale 2) in the PVs. Obs1 rated 89% of the black-blood PSIR MTC images as good image quality with artifacts in the PVs and 11% as excellent image quality without artifacts, while Obs2 rated 51% of the black-blood PSIR MTC images as good image quality with artifacts in the PVs and 49% as excellent image quality without artifacts (Fig. 4).

**Fig. 5:**
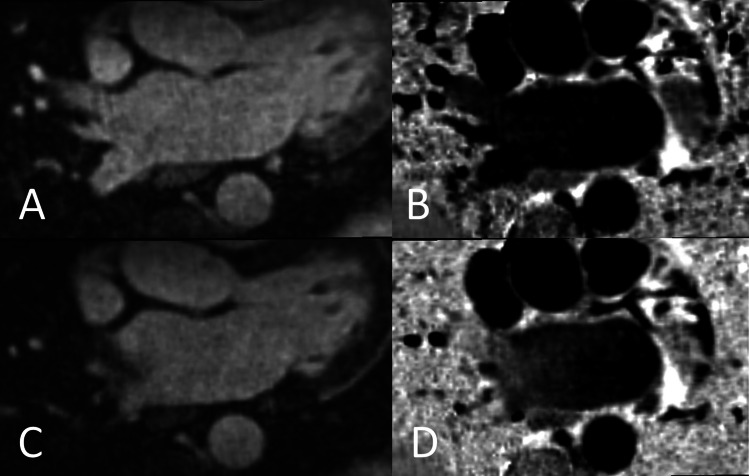
Three pulmonary veins on the right side can be observed on the MTC bright-blood magnitude (**A**) and black-blood PSIR (**B**) images. On the T2prep images (**C**, **D**), the pulmonary veins are not detectable from the same view because of inflow artifacts

PV anatomy could be assessed on MTC images in all patients. In 29 patients, normal anatomy was found with four separate PV ostia. A common trunk of the left PVs was observed in 15 cases, and three PVs on the right side were observed in 10 cases (Fig. 5). Other PV anomalies were not found in our study group. CT angiography was performed in 23 patients, showing the same anatomy as CMR. SNR and CNR measurements were not performed in the right accessory PVs. In case of common trunk of the left PVs, the SNR and CNR analyses were performed before the veins merged.

An image quality analysis was performed with respect to heart rate and presence of sinus rhythm during the measurement. In the case of sinus rhythm based on an ECG obtained within 1 h before or after the CMR examination, the observers gave a higher proportion of 3 points compared to atrial tachyarrhythmia cases (Table 2). A tendency of lower heart rates was found for cases rated with 3 points (Table 3); nevertheless, we did not find significant differences except for magnitude images with observer 1.

**Table 2:** Qualitative assessment of image quality of MTC-BOOST, and association between image quality and heart rhythm. Number of cases classified by the two observers on the Likert scale in case of sinus rhythm and atrial arrhythmia

		Obs1		Obs2	
	Likert scale	Sinus rhythm	Atrial tachyarrhythmia	Sinus rhythm	Atrial tachyarrhythmia
Magnitude	2 points	14	12	13	13
	3 points	16	3	17	2
		*p* < 0.05		*p* < 0.01	
PSIR	2 points	25	15	12	11
	3 points	5	0	18	4
		*p* = 0.10		*p* < 0.05

**Table 3:** Qualitative assessment of image quality of MTC-BOOST, and association between image quality and heart rate. Heart rate of patients divided by the rating on the Likert scale

Heart rate (bpm)				
		2 points	3 points	*p*
Obs1	Magnitude	82 ± 29	64 ± 14	< 0.05
	PSIR	77 ± 26	59 ± 8	0.08
Obs2	Magnitude	81 ± 30	66 ± 14	0.15
	PSIR	81 ± 31	68 ± 15	0.10

### Quantitative assessment of image quality of MTC-BOOST

The results of quantitative assessments of image quality showed that the SNR and CNR values of the bright-blood magnitude images were higher if patients had sinus rhythm in the majority of measurements. For some measurements, the difference was not statistically significant, but the trend was similar (Table 4).

**Table 4:** SNR and CNR values of MTC magnitude bright-blood and PSIR black-blood images according to heart rhythm

		Sinus rhythm	Atrial tachyarrhythmia	*p*
**MTC magnitude (bright-blood)—SNR**				
Obs1	LIPV	27 ± 10	21 ± 5	< 0.05
	LSPV	25 ± 7	22 ± 4	0.10
	RIPV	26 ± 8	23 ± 7	0.10
	RSPV	25 ± 7	21 ± 4	< 0.05
Obs2	LIPV	26 ± 8	20 ± 4	< 0.01
	LSPV	26 ± 8	21 ± 4	< 0.01
	RIPV	28 ± 10	21 ± 5	< 0.05
	RSPV	25 ± 8	20 ± 5	< 0.01
**MTC magnitude (bright-blood)—CNR**				
Obs1	LIPV	25 ± 10	19 ± 5	< 0.05
	LSPV	22 ± 7	19 ± 4	0.17
	RIPV	24 ± 8	21 ± 6	0.19
	RSPV	22 ± 7	18 ± 4	0.07
Obs2	LIPV	24 ± 8	18 ± 4	< 0.01
	LSPV	24 ± 8	18 ± 4	< 0.01
	RIPV	25 ± 10	19 ± 5	< 0.05
	RSPV	23 ± 8	17 ± 5	< 0.01
**MTC PSIR (black-blood)—SNR**				
Obs1	LIPV	18 ± 5	18 ± 4	0.89
	LSPV	19 ± 6	18 ± 5	0.70
	RIPV	18 ± 5	18 ± 5	0.80
	RSPV	19 ± 6	19 ± 5	0.71
Obs2	LIPV	18 ± 5	18 ± 4	0.89
	LSPV	19 ± 5	19 ± 5	0.99
	RIPV	18 ± 5	18 ± 5	0.85
	RSPV	19 ± 6	19 ± 5	0.85
**MTC PSIR (black-blood)—CNR**				
Obs1	LIPV	4.9 ± 1.8	5.3 ± 1.9	0.48
	LSPV	3.5 ± 1.2	3.9 ± 1.4	0.36
	RIPV	4.3 ± 1.5	4.4 ± 1.4	0.78
	RSPV	3.5 ± 1.1	3.8 ± 1.4	0.36
Obs2	LIPV	5.1 ± 1.6	5.3 ± 2.1	0.82
	LSPV	4.2 ± 1.6	3.9 ± 1.0	0.56
	RIPV	4.6 ± 1.6	4.7 ± 1.5	0.89
	RSPV	4.0 ± 1.2	4.1 ± 1.1	0.81

Abbreviations: *LIPV* left inferior pulmonary vein, *LSPV* left superior pulmonary vein, *RIPV* right inferior pulmonary vein, *RSPV* right superior pulmonary vein

We did not find a significant difference in SNR and CNR values between patients with sinus rhythm and atrial tachyarrhythmia on the black-blood PSIR images (Table 4).

We found a significant or nearly significant negative correlation between heart rate and the SNR and CNR values of bright-blood magnitude images. The SNR and CNR values of black-blood PSIR images did not correlate with the heart rate (Table 5).

**Table 5:** Correlations between heart rate and the SNR and CNR values of magnitude and PSIR MTC images

		Magnitude				PSIR			
		SNR		CNR		SNR		CNR	
		*r*	*p*	*r*	*p*	*r*	*p*	*r*	*p*
Obs1	LIPV	− 0.22	0.14	− 0.19	0.22	0.02	0.90	− 0.12	0.48
	LSPV	− 0.37	< 0.05	− 0.36	< 0.05	0.18	0.24	− 0.18	0.25
	RIPV	− 0.24	0.11	− 0.23	0.13	0.18	0.25	− 0.19	0.21
	RSPV	− 0.45	< 0.01	− 0.44	< 0.01	0.17	0.27	− 0.24	0.12
Obs2	LIPV	− 0.30	< 0.05	− 0.30	< 0.05	0.10	0.53	− 0.17	0.31
	LSPV	− 0.38	< 0.01	− 0.37	< 0.05	0.27	0.08	− 0.15	0.32
	RIPV	− 0.31	< 0.05	− 0.30	< 0.05	0.24	0.11	− 0.23	0.13
	RSPV	− 0.46	< 0.01	− 0.45	< 0.01	0.25	0.11	− 0.22	0.16

Abbreviations: *LIPV* left inferior pulmonary vein, *LSPV* left superior pulmonary vein, *RIPV* right inferior pulmonary vein, *RSPV* right superior pulmonary vein, *r* correlation coefficient

### Interobserver agreement

We found excellent or good agreement between the two observers in the signal intensity assessments both on the magnitude and PSIR MTC images. (Supplementary Table S2).

## Discussion

Previously, BOOST sequence was successfully applied to identify the coronary lumens, thrombi, congenital diseases, etc. MTC-BOOST outperformed the standard T2prep sequence in pulmonary vein isolations; nevertheless, this study was based on only 11 healthy volunteers and 4 patients with atrial fibrillation [8]. In our study, we focus on the feasibility of MTC measurement in clinical practice and investigate the performance of the sequence in patients with sinus rhythm and atrial tachyarrhythmia. We applied both the T2prep and MTC sequence to compare them and determine their feasibility.

The main finding of this study is that MTC-BOOST is applicable for assessing pulmonary vein independently of the patients’ heart rate and rhythm. In the study population, 30 patients had sinus rhythm and 15 patients had atrial tachyarrhythmia at the time of the CMR examination, and none of them had images of poor quality in which the PVs could not be adequately assessed. These results are very promising, especially considering the use of a fixed diastolic acquisition window (100 ms). This setting is not optimal at higher heart rates, but our experience is that the images are still well evaluated, and using this setting is comfortable and shortens the measurement time.

For MTC images, the image quality was affected by heart rate and rhythm. With subjective qualitative analysis, the observers rated images as excellent image quality with higher proportion in the case of sinus rhythm; moreover, a tendency of lower heart rate was found for cases rated as excellent image quality. The difference was significant only at bright-blood magnitude image analysis and only at observer 1.

The SNR and CNR values of bright-blood magnitude images were higher in the case of sinus rhythm. However, for some measurements, the difference was not statistically significant, but the trend was similar which can be explained by the relatively small sample size. A negative correlation was found between heart rate and the SNR and CNR values of bright-blood magnitude images in the majority of the measurements. Based on these results, the image quality of bright-blood magnitude images is influenced by the heart rate and rhythm, and it is better if patients had sinus rhythm and lower heart rate. Analyzing the black-blood PSIR images, no differences were found in the SNR and CNR values between sinus rhythm and atrial tachyarrhythmia, and the SNR and CNR values showed no correlation with the heart rate. These results suggest that the image quality of black-blood PSIR images is less affected by heart rate and rhythm.

Surprisingly, T2prep BOOST quality was under our expectation, and we found poor, non/diagnostic image quality at a large portion of the patients. We found that T2prep BOOST is not suitable for visualizing the PVs, which confirms previously noted findings [8], and is attributed to refocusing-based T2prep modules generally not working well in venous vessels (T2* deoxygenation effect) and in combination with turbulent flow (motion in the scale of T2prep refocusing times). On the other hand, it was previously demonstrated that T2prep BOOST is applicable for thrombus visualization without contrast administration [9] which has a high clinical relevance as excluding LA appendage thrombus before an electro-cardioversion or ablation therapy of AF is particularly important. However, for visualizing the LA appendage, there is no widely accepted CMR technique. With cine movies and late enhancement images, there is a risk of overlooking small but relevant LA appendage thrombi due to slice thickness and difficulties of proper angulation. Therefore, in the future, T2prep BOOST imaging may play a role in the exclusion of LA appendage thrombus.

An additional advantage of all iNAV-based BOOST techniques noted in this study is the complete absence of inflow artifacts that can occur with standard navigator techniques when a navigator acquisition crosses, e.g., pulmonary vessels. This is because the iNAV images are not generated by an additional acquisition, but instead are generated from a phase encoding of the bSSFP ramp-up pulses that would anyways be played out to bring the imaging volume into steady state [13].

## Study limitations

One limitation of our study is that it was a single-center study, which might limit the generalizability of our conclusions. Another limitation is the relatively small sample size which can explain that some of our results were statistically not significant. Furthermore, CT angiography was performed only in 23 of 45 patients. In these cases, CMR and CT showed the same PV anatomy; however, in the remaining cases, we could not validate the PV anatomy with another imaging method. Last, but not least, we think that the quantitative image analysis would be more precise if we can segment the pulmonary veins and calculate the means and standard deviations from the entire vein. Taking into account that our main aim was to investigate the clinical feasibility of BOOST imaging, we dispense from that analysis.

## Conclusion

3D whole-heart MTC-BOOST imaging is suitable for visualizing the PVs in patients with AF with excellent or good image quality, and there are no cases in the current study in which the PVs could not be adequately assessed. However, the overall image quality of bright-blood magnitude images can be influenced by the heart rate and rhythm, with slightly better image quality if patients are in sinus rhythm and have lower heart rate.

### Supplementary Information

Below is the link to the electronic supplementary material.

Supplementary file1 (PDF 85 KB)

## Abbreviations

3D: 3 Dimensional
AF: Atrial fibrillation
BOOST: Bright-blood and black-blood phase-sensitive inversion recovery
bSSFP: Balanced steady-state free precession
CMR: Cardiac magnetic resonance
CMRA: Cardiac magnetic resonance angiography
CNR: Contrast to noise ratio
ECG: Electrocardiography
FOV: Field of view
GRE: Gradient echo
ICC: Intraclass correlation coefficient
iNAV: Image navigation
IR: Inversion recovery
LA: Left atrium
LGE: Late gadolinium enhancement
MTC: Magnetization transfer
Obs: Observer
ROI: Region of interest
PSIR: Phase-sensitive inversion recovery
PV: Pulmonary vein
SD: Standard deviation
SI: Signal intensity
SNR: Signal to noise ratio
T2prep: T2 preparation

## Declarations

### Guarantor

The scientific guarantor of this publication is Hajnalka Vágó.

### Statistics and biometry

No complex statistical methods were necessary for this paper.

### Study subjects or cohorts overlap

No study subjects or cohorts have been previously reported.

### Methodology

• prospective

• observational

• performed at one institution
