# Supplementary material for: Feasibility and image quality of bright-blood and black-blood phase-sensitive inversion recovery (BOOST) sequence in clinical practice using for left atrial visualization in patients with atrial fibrillation
Source: Eur Radiol. 2023 Oct 7;34(4):2689–98. doi: 10.1007/s00330-023-10257-3 (PMC10957673; doi:10.1007/s00330-023-10257-3)
Supplement: Supplementary file 1 — Supplementary file1 (PDF 85 KB) [file 330_2023_10257_MOESM1_ESM.pdf]

Feasibility and image quality of bright-blood and black-blood phase sensitive inversion recovery (BOOST) sequence in clinical practice using for left atrial visualization in patients with atrial fibrillation

Electronic Supplementary Material

Supplementary Table S1. Summary table of imaging parameters.

	BOOST T2prep	BOOST MTC
TR (Echo spacing) [msec]	3.1	3.1
TE [msec]	1.4	1.4
Flip angle [degree]	90	90
Preparation module	T2 preparation and inversion	Magnetization transfer preparation and inversion
Inversion time TI [msec]	110	140
Average Field of View [mm]	320x320x90	320x320x90
Spatial resolution [mm]	1.4x1.4x1.4	1.4x1.4x1.4
Acquisition window [msec]	100	100
Average acquisition time [min]	6.57	7.04

Supplementary Table S2. Interobserver agreement examined with the intraclass correlation coefficient.

	ICC – MTC Magnitude (bright-blood)	ICC – MTC PSIR (black-blood)
LIPV	0.89	0.97
LSPV	0.94	0.78
RIPV	0.95	0.94
RSPV	0.99	0.89
Background mean	0.95	0.72
Background SD	0.83	0.97

Abbreviations: LIPV – left inferior pulmonary vein, LSPV – left superior pulmonary vein, RIPV – right inferior pulmonary vein, RSPV – right superior pulmonary vein, ICC –intraclass correlation coefficient, PSIR – phase sensitive inversion recovery, SD – standard deviation
